# Palmitate Enhances the Efficacy of Cisplatin and Doxorubicin against Human Endometrial Carcinoma Cells

**DOI:** 10.3390/ijms23010080

**Published:** 2021-12-22

**Authors:** Zih-Syuan Wu, Shih-Ming Huang, Yu-Chi Wang

**Affiliations:** 1 Graduate Institute of Life Sciences, National Defense Medical Center, Taipei City 114, Taiwan; g8401011@gapps.ndmctsgh.edu.tw (Z.-S.W.); shihming@ndmctsgh.edu.tw (S.-M.H.); 2 Department of Biochemistry, National Defense Medical Center, Taipei City 114, Taiwan; 3 Department of Obstetrics and Gynecology, Tri-Service General Hospital, National Defense Medical Center, Taipei City 114, Taiwan

**Keywords:** lipotoxicity, palmitate, endometrial cancer, adjuvant chemotherapy

## Abstract

Endometrial cancer is the most common gynecological cancer worldwide. At present there is no effective screening test for its early detection and no curative treatment for women with advanced-stage or recurrent disease. Overexpression of fatty acid synthase is a common molecular feature of a subgroup of sex steroid-related cancers associated with poor prognoses, including endometrial cancers. Disruption of this fatty acid synthesis leads to cell apoptosis, making it a potential therapeutic target. The saturated fatty acid palmitate reportedly induces lipotoxicity and cell death by inducing oxidative stress in many cell types. Here, we explored the effects of palmitate combined with doxorubicin or cisplatin in the HEC-1-A and RL95-2 human endometrial cancer cell lines. The results showed that physiological concentrations of exogenous palmitate significantly increased cell cycle arrest, DNA damage, autophagy, and apoptosis in both RL95-2 and HEC-1-A cells. It also increased the chemosensitivity of both cell types. Notably, we did not observe that palmitate lipotoxicity reflected increased levels of reactive oxygen species, suggesting palmitate acts via a different mechanism in endometrial cancer. This study thus provides a potential therapeutic strategy in which palmitate is used as an adjuvant in the treatment of endometrial cancer.

## 1. Introduction

Cancer of the endometrium is the most frequently occurring gynecological cancer [1]. In recent years, both the incidence of endometrial cancer and its associated mortality have been increasing rapidly around the world. In Taiwan, for example, the incidence of endometrial cancer now exceeds that of both cervical and ovarian cancer [2]. Risk factors for endometrial cancer include endometrial hyperplasia, menopausal estrogen, obesity, nulliparity, polycystic ovary syndrome, high cumulative doses of tamoxifen, diabetes, and genetic factors, among others [3]. On the basis of their clinical pathogenic mechanisms, endometrial cancers have been classified into two major types: type I is estrogen-dependent and related to hormonal imbalances, while type II is non-estrogen-dependent [4]. Most patients with endometrial cancer are type I and associated with excessive use of estrogen, endometrial hyperplasia, or obesity. Type II tumors are usually more common in obese women who may have endocrine or metabolic disorders, and they are also related to atrophic endometrium [5]. There are a number of treatments for endometrial cancer, including surgery, chemotherapy, radiation, and hormone therapy. Stage, histology, and tumor characteristics are the most important determinants guiding therapeutic strategy [6]. Statistics indicate that while most patients with low-grade tumors can be cured through surgery and chemotherapy; the survival rate among patients with advanced or recurrent endometrial cancers is very low. The main reason is resistance of the cancer cells to chemotherapy and the limited treatment methods currently available. Further research is needed to improve existing therapies to address this challenge.

Palmitate is the most abundant saturated fatty acid, accounting for 70–80% of total plasma free fatty acids. It can be supplied in food or synthesized endogenously via de novo lipogenesis (DNL) [7], which is a tightly controlled process that converts carbohydrates into the fatty acids used to synthesize triglycerides or other lipid molecules for membrane biosynthesis and energy storage [8]. Fatty acid synthase (FAS) is the key rate-limiting enzyme in DNL and converts malonyl-CoA to palmitic acid, the primary fatty acid product in DNL. Palmitate is subsequently elongated and desaturated to produce complex fatty acids, including stearic acid, palmitoleic acid, and oleic acid. In normal tissues, palmitate content is regulated to within a range of concentrations, and ingestion does not significantly affect palmitate levels in tissues [9]. The mechanism by which tissue palmitate concentrations are strictly regulated may be primarily related to maintaining normal homeostasis with tissues, including maintenance of the physical properties of membranes and the biosynthesis of palmitoylethanolamide [10]. However, under certain physiological and pathological conditions and nutritional factors, DNL may be strongly induced, increasing tissue palmitate levels and disrupting its regulation [11].

Reactive oxygen species (ROS) are the intermediates produced during the metabolic processes of organelles, such as the endoplasmic reticulum, mitochondrial respiratory complex, peroxisomes, xenobiotic detoxification, and fatty acid oxidation [12]. Mitochondria are the primary source of intracellular ROS, including hydrogen peroxide and hydroxyl radicals, that act as second messengers in cell signal transduction for different biological processes as growth, differentiation, metabolism, and apoptosis [13,14]. In normal cells, the concentration of ROS is regulated by many antioxidant systems such as peroxiredoxins, glutathione peroxidases, and catalase, therefore keeping ROS at a basal non-toxic level [15]. In cancer cells, disruption of the redox balance has been proven to be one of the most important causes of cancer occurrence, progression, and metastasis. ROS activates the cancer cell survival signal cascade, involving MAPK/ERK1/2, p38, JNK, and PI3K/Akt to activate NF-κB, matrix metalloproteinases, and VEGF to initiate cancer angiogenesis, metastasis, and survival [16]. However, there are still many controversies regarding the definition of ROS as a tumor-promoter or tumor-suppressor [17]. Palmitate exerts adverse effects in part by inducing ROS generation, which leads to lipotoxicity associated with endoplasmic reticulum stress, mitochondrial dysfunction, and cell death in a number of cell types, including adipocytes [18], glomerular podocytes [19], pancreatic β cells [20], cardiomyocytes [21], endothelial cells [22], vascular smooth muscle cells [23], and hepatocytes [24]. However, contradictory findings from one study indicate that palmitate-induced pancreatic β cell death is not caused by ROS [25]. Consequently, the mechanism by which palmitate mediates ROS production remains unclear.

Additionally, recent studies into the role of palmitate in cancer has shown that compared to normal breast and some tumor cells, HER2/neu-positive breast cancer cells show significantly increased fatty acid synthesis and storage. Moreover, when physiological doses of exogenous palmitate are added, fatty acid synthesis is disrupted, leading to CHOP (C/EBP homologous protein)-dependent apoptosis [26]. Palmitate also induces reductions in the mitochondrial membrane potential (MMP) and release of cytochrome c into the cytosol in MDA-MB-231 cells [27]. Moreover, it has been observed in patients with endometrial cancer that, compared to benign endometrial tumors, malignant tumors are enriched in glycolytic and lipogenic metabolic pathways and depend on this metabolism for survival [28]. However, the effect of palmitate in endometrial cancer remains incompletely understood.

In light of these observations, we tried to explore the relationship between high dietary fat intake and cancer risk. Our work is based on the premise that assessing the effects of individual main dietary fatty acids on endometrial cancer cells would help us understand the mechanism by which palmitate may affect tumor cells. Here, we applied physiological concentrations (10–200 μM) of exogenous palmitate and used the RL95-2 and HEC-1-A endometrial cancer cell lines to investigate the mechanism(s) underlying palmitate cytotoxicity and its effect on the cells’ responsiveness to cisplatin and doxorubicin. Our findings clarify the lipotoxicity of palmitate in endometrial cancer and have a synergistic effect with chemotherapy, providing a potential adjuvant treatment strategy.

## 2. Results

### 2.1. Palmitate Cytotoxicity in Human Endometrial Cancer Cell Lines

**Figure 1:**
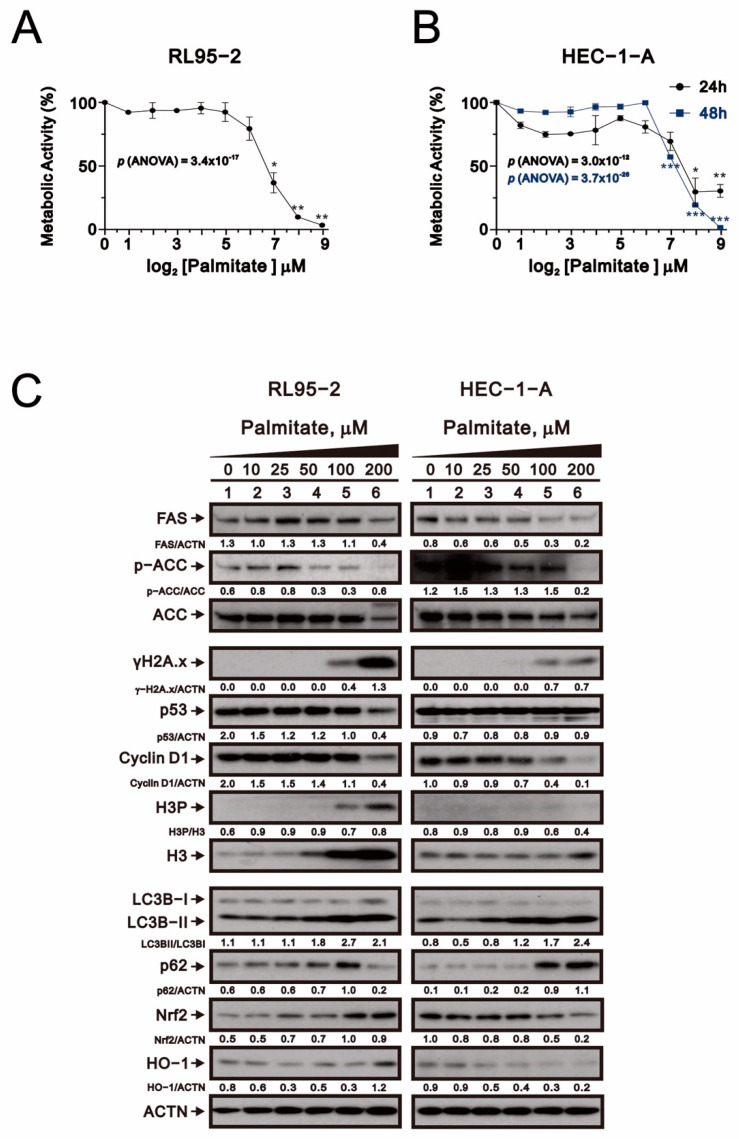
Responsiveness of human endometrial carcinoma cells to palmitate. (**A**,**B**) Metabolic activity measured using the MTT method. RL95-2, and HEC-1-A cells were treated for 24 h or 48 h with palmitate (0, 1.953125, 3.90625, 7.8125, 15.625, 31.25, 62.5, 125, 250, 500 μM). Symbols depict the mean ± SD of three independent experiments. * *p* < 0.05, ** *p* < 0.01, and *** *p* < 0.001 (Student’s *t*-tests). (**C**) RL95-2, and HEC-1-A cells were treated for 24 h with indicated concentrations of palmitate. Cell lysates were subjected to Western blot analysis using antibodies against the indicated proteins. Alpha actinin (ACTN) was the loading control.

We first examined the metabolic activity of palmitate toward the RL95-2 (type I) and HEC-1-A (type II) endometrial cancer cell lines. The two cell lines were incubated for 24 or 48 h with increasing doses of palmitate, and metabolic activity was detected with MTT conversion assays (Figure 1A,B). Our preliminary data showed that the ED50 for palmitate toxicity was estimated to be 69.51 μM in RL95-2 treated for 24 h and 56.89 μM for HEC-1-A cells treated for 48 h.

To better understand the mechanism underlying palmitate cytotoxicity, we examined its effect on the levels of various proteins. Western blot analysis showed that levels of FAS, p53, cyclin D1 (a cell cycle G1 biomarker), and the ratio of phosphorylated to total ACC (fatty acid synthesis biomarker) were all significantly and dose-dependently decreased in both RL95-2 and HEC-1-A cells (Figure 1C). The H3P/H3 ratio, a cell cycle G2/M biomarker, was difficult to determine because H3 and H3P proteins were both dose-dependently elevated in RL95-2 cells. At higher doses, palmitate also induced expression of γH2A.x (a DNA damage biomarker) and dose-dependently increased p62 levels and the LC3B II/I ratio (two autophagy biomarkers). The effect of palmitate on levels of Nrf2 and HO-1 (anti-ROS stress biomarkers) differed between RL95-2 and HEC-1-A cells; although their levels were dose-dependently increased in RL95-2 cells, they were decreased in HEC-1-A cells. Overall, responsiveness to palmitate was more apparent in RL95-2 than HEC-1-A cells.

### 2.2. Synergistic Effects of Palmitate and Chemotherapeutic Drugs in Human Endometrial Cancer Cell Lines

**Figure 2:**
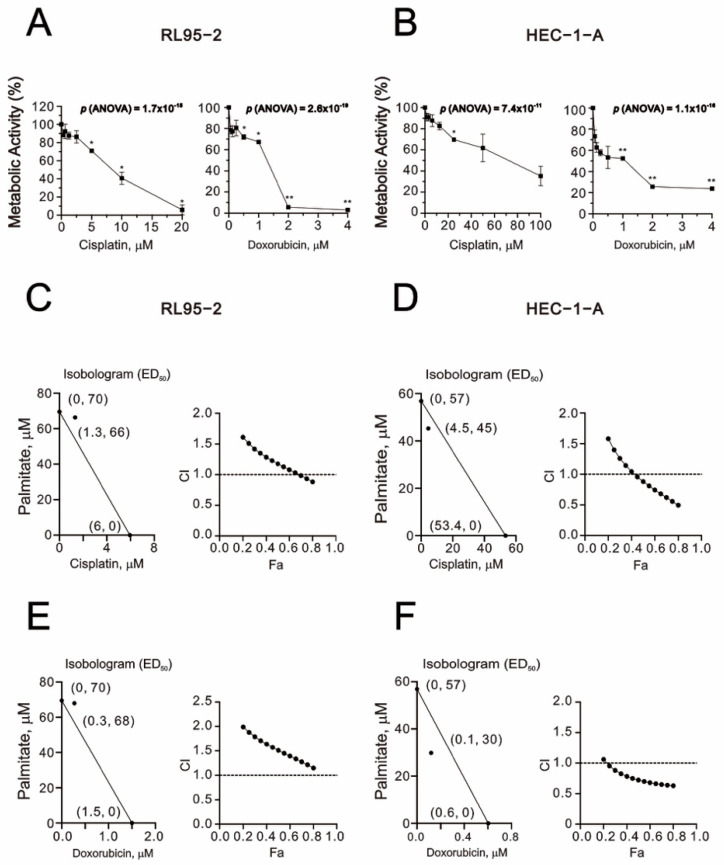
Combination indexes for palmitate with cisplatin or doxorubicin in RL95-2 and HEC-1-A cells. (**A**,**B**) Metabolic activity measured using the MTT method. RL95-2, and HEC-1-A cells were treated with doxorubicin (0, 0.0625, 0.125, 0.25, 0.5, 1, 2, 4 μM) or cisplatin (0, 0.3125, 0.625, 1.25, 2.5, 5, 10, 20 μM), (0, 1.5625, 3.125, 6.25, 12.5, 25, 50, 100 μM), respectively. Symbols depict the mean ± SD of three independent experiments. * *p* < 0.05, ** *p* < 0.01 (Student’s *t*-tests). (**C**,**E**) RL95-2 cells were treated for 24 h with palmitate (0, 1.953125, 3.90625, 7.8125, 15.625, 31.25, 62.5, 125, 250, 500 μM) combined with cisplatin (0, 0.3125, 0.625, 1.25, 2.5, 5, 10, 20 μM) or doxorubicin (0, 0.0625, 0.125, 0.25, 0.5, 1, 2 μM). (**D**,**F**) HEC-1-A were treated for 48 h with palmitate (0, 1.953125, 3.90625, 7.8125, 15.625, 31.25, 62.5, 125, 250, 500 μM) combined with cisplatin (0, 1.5625, 3.125, 6.25, 12.5, 25, 50, 100 μM) or doxorubicin (0, 0.0625, 0.125, 0.25, 0.5, 1, 2 μM). Metabolic activity was measured using the MTT method. Isobolograms (ED_50_) were calculated using CalcuSyn 2.0 software.

To test whether palmitate has synergistic effects with conventional chemotherapeutic agents, we applied the Chou–Talalay method to calculate the CIs and dosage requirements of palmitate with cisplatin or doxorubicin in RL95-2 and HEC-1-A cells. When administered individually in RL95-2, the IC50 values for cisplatin or doxorubicin for 24 h were about 6.0 and 1.5 μM. In HEC-1-A, the IC50 values for cisplatin or doxorubicin for 48 h were about 53.4 and 0.6 μM (Figure 2A,B). In combination with palmitate and cisplatin, the CI was <1 in both RL95-2 and HEC-1-A cells, indicating synergistic effects (Figure 2C,D). On the other hand, for palmitate with doxorubicin, the CI was <1 only in HEC-1-A cells (Figure 2E,F). A significant synergistic effect was observed when palmitate was combined with cisplatin at concentrations ranging from 4.5 μM to 53.4 μM (Figure 2D).

### 2.3. Molecular Mechanisms of Palmitate and Chemotherapeutic Drugs in Human Endometrial Cancer Cell Lines

**Figure 3:**
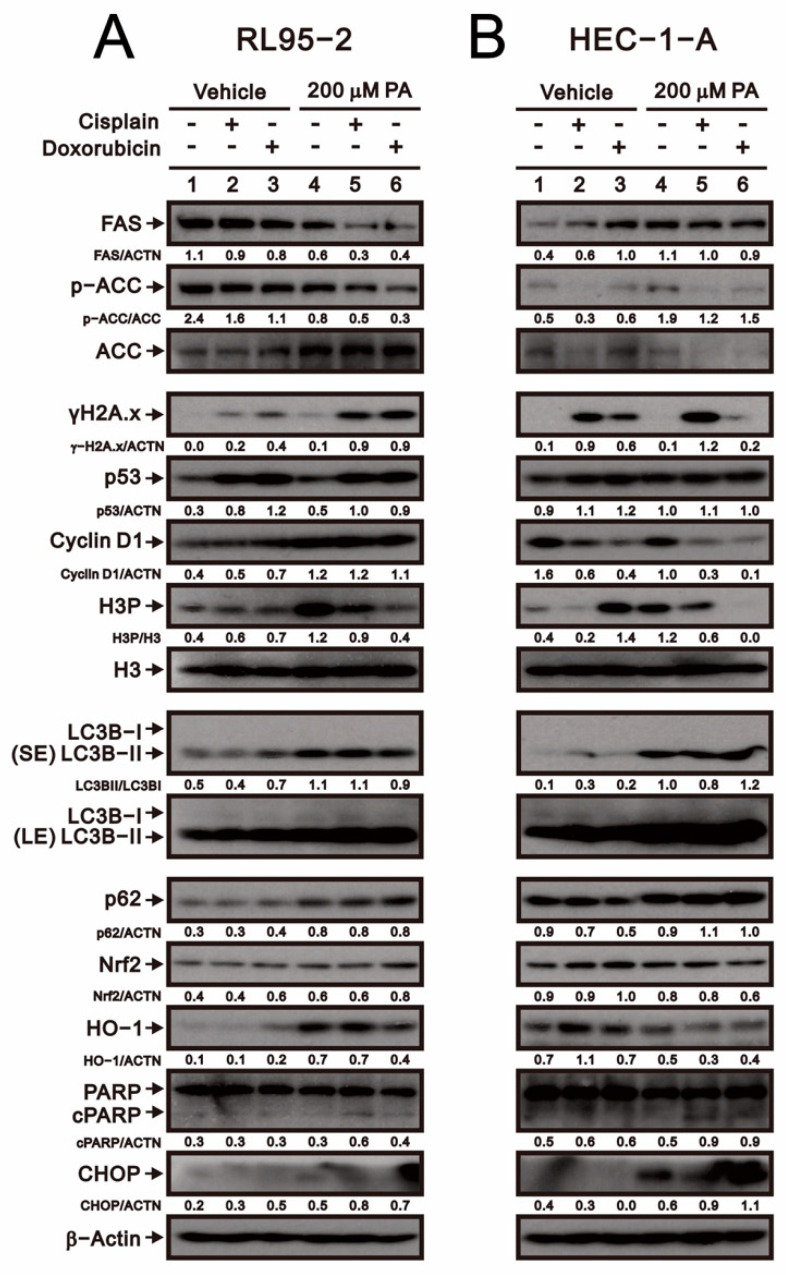
Effects of palmitate with cisplatin or doxorubicin on protein expression in RL95-2 and HEC-1-A cells. (**A**) RL95-2 cells were incubated for 24 h with 200 μM palmitate plus 5 μM cisplatin or 0.5 μM doxorubicin. (**B**) HEC-1-A cells were incubated for 24 h with 200 μM palmitate plus 50 μM cisplatin or 0.5 μM doxorubicin. Cell lysates were subjected to Western blot analysis using antibodies against the indicated proteins. Beta-actin was the loading control.

We next investigated the effects of combination therapy on levels of various proteins in RL95-2 and HEC-1-A cells. In RL95-2 cells, cisplatin and doxorubicin individually increased γH2A.x, p53, and H3, and their abilities to increase levels of γH2A.x, p53, LC3B, CHOP, and cleaved PARP were all enhanced by palmitate (Figure 3A). Conversely, palmitate downregulated the effects of cisplatin and doxorubicin on FAS, HO-1, and the p-ACC/ACC and H3P/H3 ratios in RL95-2 cells. In HEC-1-A cells, palmitate downregulated the effects of cisplatin and doxorubicin on p53 and the p ACC/ACC and H3P/H3 ratios, whereas it enhanced their effects on LC3B, CHOP, and cleaved PARP (Figure 3B). These results indicate that the mechanisms by which palmitate acts in combination with chemotherapy drugs differ in RL95-2 and HEC-1-A cells.

### 2.4. Effects of Palmitate and Chemotherapeutic Drugs on the Cell Cycle Profile, Cellular Proliferation, and Apoptosis in Human Endometrial Cancer Cell Lines

**Figure 4:**
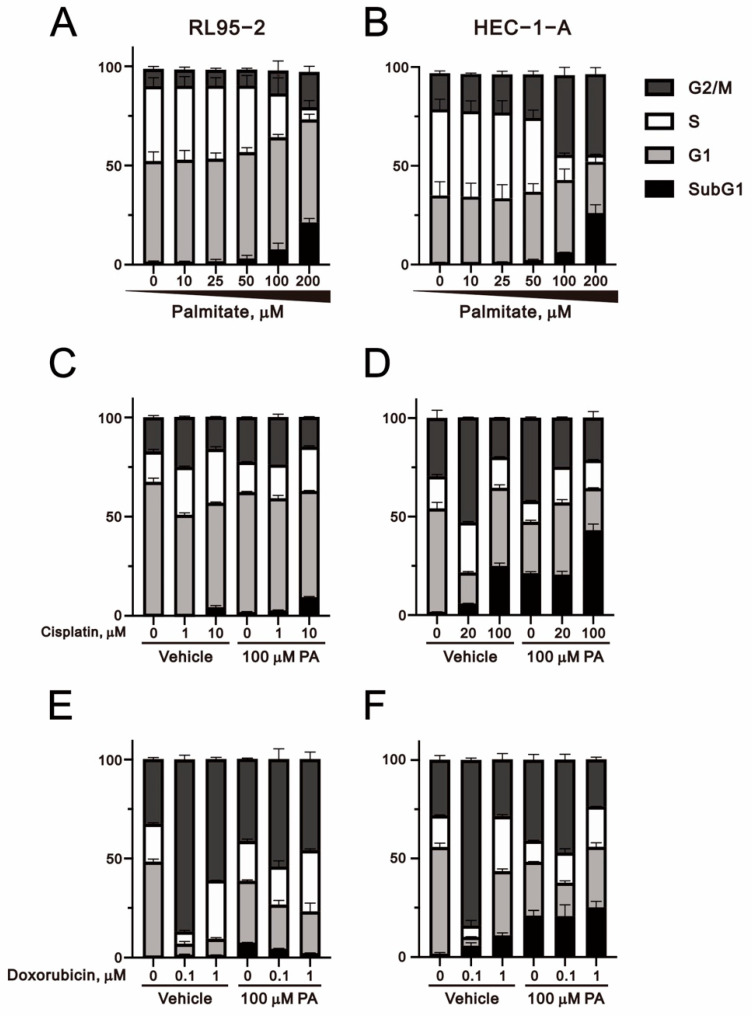
Effect of palmitate alone and in combination with cisplatin or doxorubicin on the cell cycle profiles in human endometrial cancer cells. (**A**,**C**,**E**) RL95-2 and (**B**,**D**,**F**) HEC-1-A cells were incubated for 24 h, after which they were treated with the indicated concentrations of doxorubicin or cisplatin plus 100 μM palmitate for 24 or 48 h. Cell cycle profiles were then analyzed using flow cytometry. Bars depict the mean ± SD of three independent experiments.

Because palmitate induced downregulation of cyclin D1, a cell cycle-related protein, in both RL95-2 and HEC-1-A cells and also significantly induced H3 and H3P (a cell cycle G2/M biomarker) in RL95-2 cells (Figure 1B), we used PI staining to test the effect of palmitate on cell cycle profiles in RL95-2 and HEC-1-A cells. In both RL95-2 and HEC-1-A cells, palmitate significantly induced cell cycle arrest in the subG1 and G2/M phases and inhibited the S phase (Figure 4A,B). Moreover, combining cisplatin or doxorubicin with palmitate enabled us to verify the cell cycle changes they reportedly induce in cancer cells (Figure 4C,D for cisplatin; Figure 4E,F for doxorubicin). It is well known that cisplatin works on G1 populations [29], while doxorubicin works on the G2/M population [30]. We found that in RL95-2 cells, combined treatment with palmitate and cisplatin increased the population at sub-G1 phase, whereas the combined treatment with doxorubicin decreased the sub-G1 phase population. In HEC-1-A cells, for palmitate in combination with cisplatin or doxorubicin, the sub-G1 phase tended to increase, which is consistent with the results summarized in Figure 2 and Figure 3 and points to a synergistic effect of the combined therapy in HEC-1-A cells.

**Figure 5:**
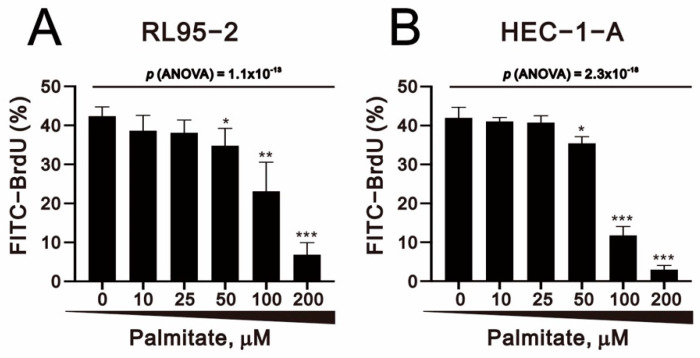
Effect of palmitate on proliferation of human endometrial cancer cells. (**A**) RL95-2 and (**B**) HEC-1-A cells were incubated for 24 h, after which they were treated for 24 or 48 h with the indicated concentrations of palmitate. Cell proliferation indicated by BrdU incorporation was analyzed using flow cytometry. Bars depict the mean ± SD of three independent experiments. * *p* < 0.05, ** *p* < 0.01, and *** *p* < 0.001 (Student’s *t*-tests).

Given the finding that palmitate significantly reduced the numbers of cells at S-phase (Figure 4A,B), we used BrdU staining to assess palmitate effects on cell proliferation. Our results showed that palmitate significantly and dose-dependently reduced cell proliferation capability in RL95-2 and HEC-1-A cells (Figure 5A,B).

**Figure 6:**
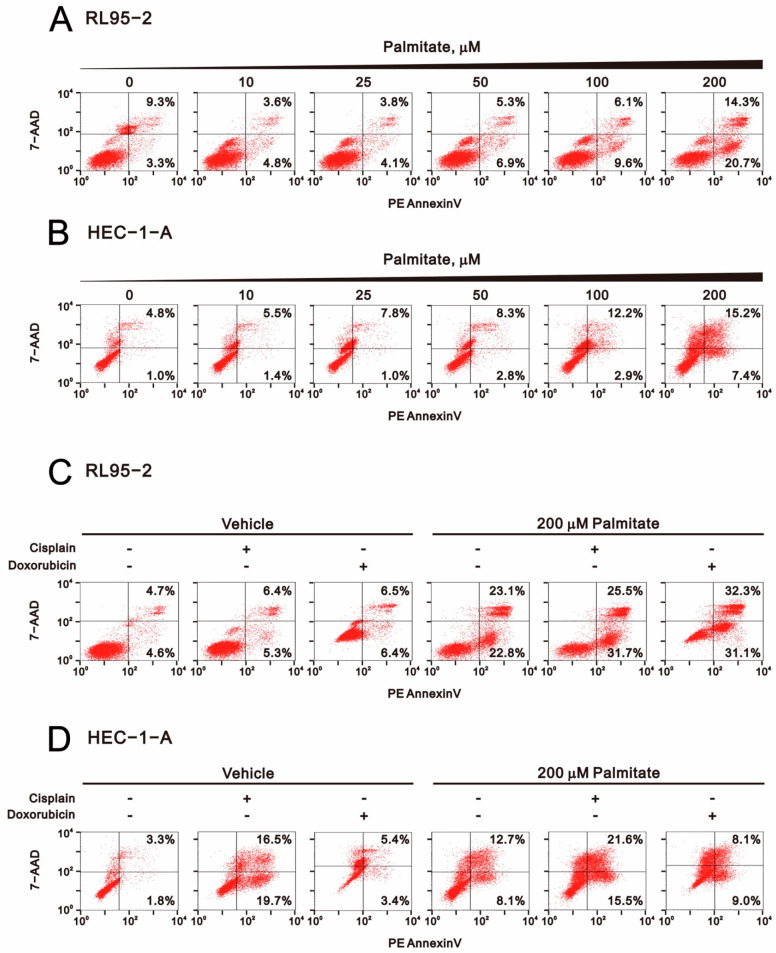
Effect of palmitate alone and in combination with cisplatin or doxorubicin on apoptosis among human endometrial cancer cells. (**A**) RL95-2 and (**B**) HEC-1-A cells were incubated for 24 h, after which they were treated for 24 or 48 h with palmitate (0, 10, 25, 50, 100, 200 μM). (**C**) RL95-2 cells treated for 24 h with 5 μM cisplatin or 0.5 μM doxorubicin plus 200 μM palmitate. (**D**) HEC-1-A cells treated for 48 h with 50 μM cisplatin or 0.5 μM doxorubicin plus 200 μM palmitate. Apoptosis markers labeled by PE-Annexin V and 7-AAD were analyzed using flow cytometry.

To determine whether palmitate-induced cytotoxicity and the elevation in sub-G1 populations leads to increased apoptosis among RL95-2 and HEC-1-A cells, we used Annexin V-PE and 7-AAD labelling to quantitatively assess cellular apoptosis. After palmitate treatment, the early and late apoptotic cell populations were increased significantly among both RL95-2 and HEC-1-A cells (Figure 6A,B). Cisplatin and doxorubicin each increased the early and late apoptotic populations among RL95-2 cells, but cisplatin only increased the early and late apoptotic populations in HEC-1-A cells (Figure 6C,D). Synergistic effects on total (early plus late) apoptotic populations were observed when RL95-2 and HEC-1-A cells were treated with palmitate plus cisplatin or doxorubicin. In RL95-2 cells, the synergistic effect with cisplatin was in the early apoptotic population, while the synergistic effect with doxorubicin was in both the early and late apoptotic populations. In HEC-1-A cells, the synergistic effect with cisplatin was in the late apoptotic population, while the synergistic effect with doxorubicin was in the early and late apoptotic populations.

### 2.5. Effects of Palmitate and Chemotherapeutic Drugs on Mitochondrial Function in Human Endometrial Cancer Cell Lines

**Figure 7:**
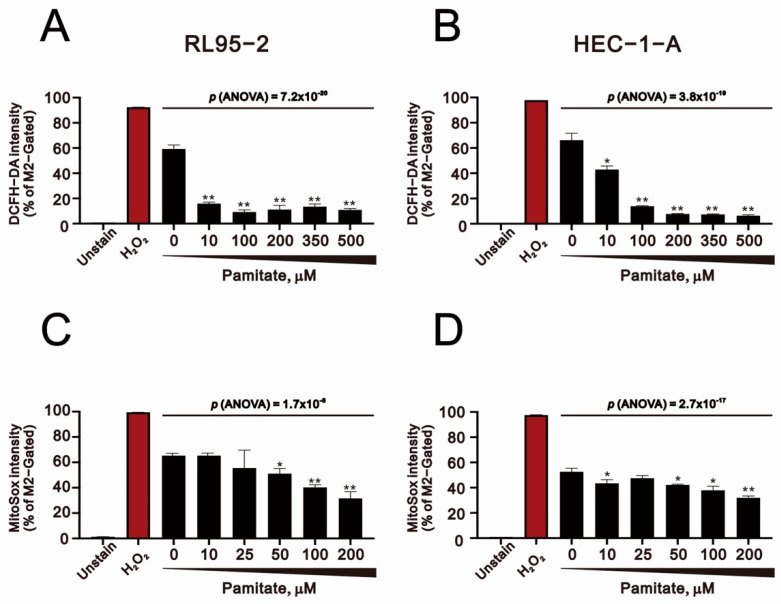
Effects of palmitate on ROS levels in human endometrial cancer cells. (**A**) RL95-2 and (**B**) HEC-1-A cells were incubated for 24 h, after which they were treated for 1.5 h with the indicated concentration of palmitate. Cellular ROS levels were monitored using 10 μM DCFH-DA with flow cytometry. (**C**) RL95-2 and (**D**) HEC-1-A cells were incubated for 24 h, after which they were treated for 1.5 h with the indicated concentration of palmitate. Mitochondrial ROS levels were monitored using 5 μM MitoSOX with flow cytometry. H_2_O_2_ serves as a positive control in all panels. Bars depict the mean ± SD of three independent experiments. * *p* < 0.05, ** *p* < 0.01 (Student’s *t*-tests).

Mitochondria play key roles in cellular survival, ROS generation, and stress-induced programmed cell death. Moreover, recent studies suggest ROS are involved in palmitate-induced apoptosis [31,32,33]. We therefore assessed ROS levels in palmitate-treated RL95-2 and HEC-1-A cells. We found that whether using DCFH-DA to measure overall cell ROS levels or MitoSox to measure mitochondrial superoxide, ROS levels declined as the palmitate concentration increased in both RL95-2 and HEC-1-A cells (Figure 7A–D). H_2_O_2_, which served as a positive control, significantly increased ROS levels in our two assays.

**Figure 8:**
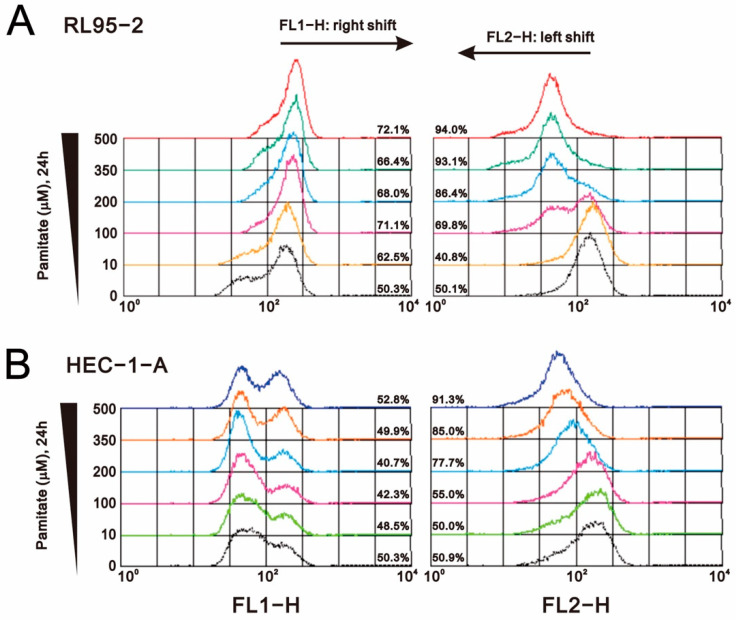
Effects of palmitate on mitochondrial membrane potential in human endometrial cancer cells. (**A**) RL95-2 and (**B**) HEC-1-A cells were incubated for 24 h, treated for 24 h with the indicated concentration of palmitate, and stained for 15 min with JC-1 dye. Mitochondrial membrane potential was detected using flow cytometry. Traces shown are representative of three independent experiments.

Because loss of MMP is a hallmark of apoptosis activation [34], we used JC-1 dye to measure changes in MMP after palmitate treatment in RL95-2 and HEC-1-A cells (Figure 8A,B). The results revealed that palmitate dose-dependently reduced MMP in both RL95-2 and HEC-1-A cells.

**Figure 9:**
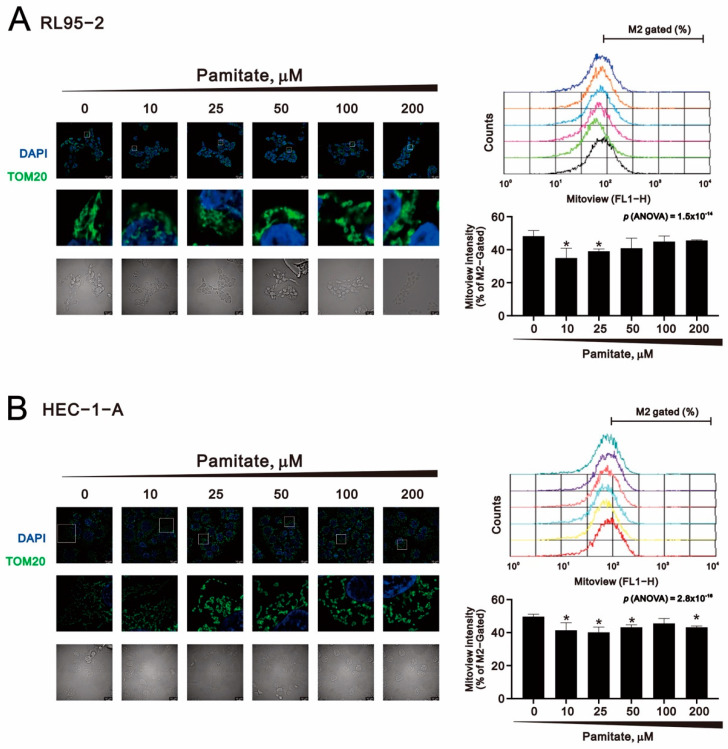
Effects of palmitate on mitochondrial morphology in human endometrial cancer cells. (**A**) RL95-2 and (**B**) HEC-1-A cells were treated for 1.5 h with the indicated concentrations of palmitate, after which they immunostained for TOM20 (mitochondria, green). Nuclei were stained with 4′,6-diamidino-2-phenylindole (DAPI, blue). Images were obtained using a Leica THUNDER Imager microscope (100x oil-immersion objective). Scale bar: 10 μm. Mitochondrial mass was assayed using MitoView^TM^ Green with flow cytometry. Traces are representative of three independent experiments. Bars depict the mean ± SD of three independent experiments. * *p* < 0.05 (Student’s *t*-tests).

Mitochondrial morphology is dynamic, as the organelles continually undergo fission and fusion in response to their environmental conditions [35]. When cells sense mild stress, mitochondria form an elongated and interconnected network to increase ATP production. Under severe cellular stress, however, mitochondria divide into fragments for mitophagy or apoptosis. MitoView ^TM^ Green is a green fluorescent mitochondrial dye, the signal from which is based on mitochondrial mass rather than mitochondrial membrane potential. We used immunofluorescent staining with TOM20 and MitoView^TM^ Green to evaluate the effect of palmitate on mitochondrial morphology and mass in RL95-2 and HEC-1-A cells (Figure 9A,B). The results showed that palmitate not only triggered mitochondrial fragmentation in both RL95-2 and HEC-1-A cells, but they also decreased mitochondrial mass. These findings suggest that palmitate causes mitochondrial damage in RL95-2 and HEC-1-A cells.

## 3. Discussion

In this work, we found that palmitate may increase the sensitivity of endometrial cancer cells to chemotherapy drugs. Our findings suggest it significantly increased cell cycle arrest, DNA damage, autophagy, and apoptosis in RL95-2 and HEC-1-A cells, with RL95-2 cells being more sensitive to palmitate than HEC-1-A cells. An earlier study of the metabolic profiles of seven endometrial cancer cell lines revealed that RL95-2 and HEC-1-A cells depend on different metabolic pathways [28]. The extracellular acidification rate; oxidation of glucose, glutamine, and palmitate; and DNL from glucose, glutamine, and acetate were all higher in RL95-2 than HEC-1-A cells. Notably, RL95-2 cells were the most dependent on DNL pathways among the seven endometrial cancer cell lines tested. We suggest that this may explain why RL95-2 cells are more sensitive to palmitate than are HEC-1-A cells.

The overall purpose of this study was to investigate the impact and underlying mechanisms of palmitate in two types of endometrial cancer. Known as the “triple endometrial cancer syndrome,” obesity, diabetes, and hypertension often co-exist in patients with endometrial cancer [36]. Recent studies indicate that palmitate levels are increased in cerebrospinal fluid from overweight and obese individuals and correlate positively with body mass index and abdominal circumference [37]. Elevation of palmitate in cerebrospinal fluid was also seen in overweight people with diabetes, dyslipidemia, and/or hypertension. One study reported that enzymes catalyzing glycolysis and DNL, including ACC1, ACC2, and FAS, are upregulated in most endometrial tumor tissues as compared to adjacent nonmalignant tissues [28]. Overexpression of FAS has been detected during the early stages of cancer development, is more pronounced in more advanced tumors, and is typically associated with a poor prognosis [38]. Recent findings have also shown that FAS blockade decreases cell proliferation and viability by stimulating apoptosis in endometrial carcinoma cells [39]. In addition to FAS, our present findings demonstrate that palmitate directly downregulates ACC and H3 protein levels. Details of the mechanisms remain to be investigated in the future.

Results from several studies have led to differing conclusions about the properties of palmitate. Several studies have reported that palmitate exhibits potential tumorigenic properties [40,41,42], although they also reported that it exhibits anticancer activity. In terms of promoting cancer, evidence suggests that palmitate may increase carcinogenesis by regulating DNA damage and inflammation [43], induce invasion of pancreatic cancer cells through TLR4/ROS/NF-κB/MMP-9 pathway [44], and increase colorectal cancer cell proliferation in a β2-adrenergic receptor-dependent manner [45]. Regarding anticancer activity, palmitate reduced cell membrane fluidity and limited glucose metabolism to enhance the anticancer effect of methylseleninic acid in hepatocellular carcinoma cells [46] and induced cell cycle G2/M arrest and promoted apoptosis in human neuroblastoma cells and breast cancer cells [47,48]. Moreover, in breast cancer, it has been observed that palmitate induced a different transcription program, reducing expression of HER2 and HER3, thereby sensitizing the cells to trastuzumab [26]. Palmitic acid isolated from the red alga Amphiroa zonata has anti-tumor activity both in vitro and in vivo. Amazingly, palmitic acid showed selective toxicity and induced apoptosis in leukemia cell lines but showed very low cytotoxicity to normal cell lines used as controls in the study [49]. Furthermore, in one study, palmitic acid is expected to become a novel anticancer agent for the treatment of prostate cancer. Their research showed that palmitic acid may inhibit the tumor metastasis regulator protein in human prostate cancer cells by inhibiting the PI3K/Akt pathway to induce cell cycle G1 phase arrest and anti-metastatic efficiency [50]. Due to the diversity of cancer phenotypes, involving factors are complicated in terms of defining palmitate as a tumor-promoter or tumor-suppressor. The working model of endometrial cancer in this study is one of the cancers that has been reported to overexpress FAS, which have developed peculiar metabolic pathways to gain a greater glucose uptake and to synthetize endogenous fatty acids. Our results indicated that palmitate plays a role in the inhibition of endometrial cancer cells. Furthermore, we used palmitate as an adjuvant to increase the sensitivity of endometrial cancer cells to chemotherapy drugs, cisplatin and doxorubicin, for the development a potential therapeutic strategy of endometrial cancer treatment.

Many literatures pointed out that the lipotoxicity induced by palmitate is characterized by the accumulation of intracellular ROS, which leads to an increase in oxidative stress and ultimately to cell apoptosis [7,51,52,53]. The effect of lipo-apoptosis caused by ROS seems to be cell type-dependent, including adipocytes, glomerular podocytes, pancreatic β cells, cardiomyocytes, endothelial cells, vascular smooth muscle cells, and hepatocytes. In contrast, apoptosis of palmitate-treated neonatal cardiomyocytes is independent of oxidative stress, and ROS is not the primary cause of palmitate-induced pancreatic β cell death [25]. It has been reported that redox homeostasis appears to be a key factor in the normal function of mitochondria and organisms. Both high levels of ROS (oxidative stress) and too low levels of ROS (reductive stress) are harmful and apparently play a pathogenic role. It has been shown that palmitate stimulated the function of UCP 1 in mitochondria, which can reduce the generation of ROS by mild uncoupling [54,55]. Moreover, the amphiphile nature of free fatty acids promotes their incorporation into the inner mitochondrial membrane, which leads to changes in membrane fluidity [56]. Consequently, it is necessary to verify whether palmitate-induced superoxide directly activates the UCP, which leads to negative feedback controlling both ROS production and their levels.

Autophagy also contributes to the reduction of ROS levels under various stress conditions [57]. The level of ROS may play an important role in regulating the formation of autophagy through various signaling pathways, including FOXO3–LC3/BNIP3 and NRF2–P62 pathways [58]. ROS produced by damaged mitochondria may induce mitophagy and eliminate damaged organelles to reduce ROS levels. The loss of mitochondrial membrane potential was initially considered to be a clue to mitophagy [59]. While palmitate was not observed to increase ROS in our study, palmitate treatment demonstrated the loss of mitochondrial membrane potential, triggered mitochondrial fragmentation, and decreased mitochondrial mass in both RL95-2 and HEC-1-A cells. Either alone or in combination, palmitate has been showed to increase the protein levels of p62 and LC3B II. These findings suggest that mitophagy might be the cause of the decline in ROS levels in RL95-2 and HEC-1-A cells. The detailed mechanism remains to be investigated in the future.

## 4. Materials and Methods

### 4.1. Cell Culture and Reagents

The RL95-2 (ATCC^®^CRL-1671™) and HEC-1-A (ATCC^®^HTB-112™) human endometrial carcinoma cell lines were purchased from the American Type Culture Collection (Manassas, VA, USA). RL95-2 cells were cultured in Dulbecco’s modified Eagle’s medium nutrient mixture F-12 (DMEM/F12) supplemented with 10% fetal bovine serum (FBS), 0.005 mg/mL insulin, and 1% penicillin–streptomycin (Thermo Fisher Scientific, Waltham, MA, USA). HEC-1-A cells were cultured in McCoy’s 5A medium supplemented with 10% FBS and 1% penicillin–streptomycin. Doxorubicin, cisplatin, sodium palmitate, 2′,7-dichlorofluorescein diacetate (DCFH-DA), propidium iodide (PI), and thiazolyl blue tetrazlium bromide (MTT) were obtained from Sigma Aldrich (St. Louis, MO, USA).

### 4.2. Analysis of Cell Metabolic Activity

RL95-2 (8 × 10^3^) and HEC-1-A (5 × 10^3^) cells were seeded into 96-well plates and incubated in their respective media. The next day, they were exposed to the indicated drugs in fresh DMEM/F12 or McCoy’s 5A medium for the indicated periods. MTT solution (0.5 mg/mL in phosphate-buffered saline (PBS)) was then added to each well, and the cells were incubated for 4 h at 37 °C. After removing the supernatants, we added dimethyl sulfoxide (DMSO; 100 μL) to dissolve the precipitate, and the absorbances at 570 nm and 650 nm were then measured using an enzyme-linked immunosorbent assay plate reader (Multiskan EX, Thermo Fisher Scientific). The relative metabolic activity was calculated on the basis of the absorbance ratio between cells cultured with the indicated drugs and the untreated controls, which were assigned a value of 100. A combination index (CI) was also calculated using CalcuSyn 2.0 software (Biosoft, Cambridge, United Kingdom) to produce an isobologram where a CI < 1 indicates a synergistic combination effect and a CI > 1 indicates an antagonistic combination effect [60].

### 4.3. Western Blot Analysis

RL95-2 and HEC-1-A cells were lysed in radio-immunoprecipitation assay buffer (100 mM Tris-HCl (pH 8.0), 150 mM NaCl, 0.1% SDS, and 1% Triton 100) at 4 °C. Proteins in the resultant lysates were separated by sodium dodecyl sulfate polyacrylamide gel electrophoresis, after which the resolve proteins were immunoblotted with antibodies against β-actin, p53, p62, FAS, nuclear factor-erythroid factor 2-related factor 2 (Nrf2) (Santa Cruz Biotechnology, Santa Cruz, CA, USA), phospho-histone H3 (H3P; serine phosphorylation at residue 10), histone H3 (H3), microtubule-associated proteins 1A/1B light chain 3B (LC3B), phospho-acetyl-CoA carboxylase (p-ACC; serine phosphorylation at residue 79), ACC, cleaved poly-ADP-ribose polymerase (cPARP), CHOP (Cell Signaling, Danvers, MA, USA), phospho-histone H2A.X (γH2A.X; serine phosphorylation at residue 139), Cyclin D1 (Abcam, Cambridge, United Kingdom), and heme oxygenase 1 (HO-1) (Enzo Life Sciences, Farmingdale, NY, USA).

### 4.4. Cell Cycle Profiles and Cellular Proliferation Analysis

RL95-2 (6 × 10^5^) and HEC-1-A (3 × 10^5^) cells were seeded into 6-well plates in their respective media. The next day, the indicated drugs were added in fresh medium, and the cells were incubated for an additional 24 or 48 h. For cell cycle analysis, the cells were fixed in 70% ice-cold ethanol and stored at −20 °C overnight. The fixed cells were then centrifuged (1000 rpm, 5 min), washed twice with ice-cold PBS supplemented with 1% FBS, and stained with PI solution (5 μg/mL PI in PBS, 0.5% Triton X-100, and 0.5 μg/mL RNase A) for 30 min at 37 °C in the dark. For each condition, 10,000 cells were analyzed using a BD FACScalibur™ flow cytometer and Cell Quest Pro software (version 5.1) (BD Biosciences, Franklin Lakes, NJ, USA).

For cell proliferation assays, following the incubation protocol described above, the cells were incubated for an additional 1 h with 10 μM BrdU (BD Pharmingen BrdU Flow Kit, San Diego, CA, USA). The medium was then discarded, and the cells were fixed at room temperature for 30 min and treated with FITC-conjugated anti-BrdU antibody (BD Pharmingen). After washing, the cells were incubated with 7-AAD and analyzed using a BD FACSCalibur™ flow cytometer and CellQuest Pro software (BD Biosciences).

### 4.5. Apoptosis Analysis

For apoptosis assays, the cells were stained with Annexin V-PE and 7-AAD and then detected with flow cytometry using the manufacturer’s protocol (BD PharMingen, San Diego, CA, USA). Briefly, after treatment with the indicated drugs, cells were washed twice with ice-cold PBS and stained with 5 µL of Annexin V-PE and 10 µL of 7-AAD (5 µg/mL) in 1 mL of binding buffer for 15 min at room temperature in the dark. Apoptotic cells were then counted using a BD FACSCalibur™ flow cytometer and Cell Quest Pro software (BD Biosciences, Franklin Lakes, NJ, USA).

### 4.6. ROS Analysis

The DCFH-DA fluorescent marker was used to identify intracellular ROS levels. In addition, MitoSOX™ Red (Invitrogen, Carlsbad, CA, USA) is a fluorescent dye that reacts selectively with mitochondrial superoxide in live cells. Cells were incubated for 1.5 h with selected doses of palmitate and then stained with DCFH-DA (10 μM) or MitoSOX™ Red (10 μM) in serum-free medium for 30 min at 37 °C and harvested. Samples were then evaluated using a FACSCalibur flow cytometer and Cell Quest Pro software (BD Biosciences, Franklin Lakes, NJ, USA).

### 4.7. Mitochondrial Membrane Potential Analysis

RL95-2 (6 × 10^5^) and HEC-1-A (3 × 10^5^) cells were seeded into 6-well plates in their respective media. The next day, selected doses of palmitate were added in fresh medium, and the cells were incubated for an additional 24 or 48 h. All dead and viable cells were then harvested, washed with PBS, and incubated with 1× binding buffer containing the MMP-sensitive fluorescent dye JC-1 for 30 min at 37 °C in the dark. The cells were then washed twice with PBS, resuspended in 500 µL of 1× binding buffer, and analyzed using a FACSCalibur flow cytometer and Cell Quest Pro software (BD Biosciences, Franklin Lakes, NJ, USA). Mitochondrial depolarization was measured on the basis of a decrease in the red/green fluorescence intensity ratio.

### 4.8. Immunocytochemistry

Immunocytochemical analysis was carried out with cells adhering to cover slips in 24-well plates. After treatment with selected doses of palmitate for 1.5 h, the cells were fixed for 10 min in 4% formaldehyde, incubated for 10  min in 0.1% Triton X-100 solution, washed 3 times in PBS, and treated for 1 h at room temperature with 1% BSA. Thereafter, the cells were incubated with anti-TOM20 antibody at 4 °C overnight. The next day, the cells were washed with PBS and incubated for 1 h with FITC. Cell nuclei were stained by DAPI. Mitochondrial morphology was observed using a THUNDER Imager microscope equipped with a 100× objective (Leica, Wetzlar, Germany).

### 4.9. Mitochondrial Mass Assay

RL95-2 (6 × 10^5^) and HEC-1-A (3 × 10^5^) cells were seeded into 6-well plates in their respective media. The next day, the cells were treated with selected doses of palmitate for 1.5 h and stained with 20 µM MitoView^TM^ Green (Biotium, CA, USA) for 30 min at 37 °C in the dark. Samples were then evaluated using a FACSCalibur flow cytometer and Cell Quest Pro software (BD Biosciences, Franklin Lakes, NJ, USA).

### 4.10. Statistical Analysis

Values were expressed as the mean ± SD from at least three independent experiments. All comparisons between groups were made using Student’s *t*-tests. Statistical significance: n.s., not significant, * *p* < 0.05; ** *p* < 0.01; *** *p* < 0.001. The comparison between multiple groups was conducted using analysis of variance (ANOVA). Statistical significance was set at *p* < 0.05.

## 5. Conclusions

Our findings indicate that palmitate exhibits potential anti-endometrial cancer activity, especially in HEC-1-A cells, which are type II endometrial cancer cells and therefore less sensitive to chemotherapy. Palmitate administered as an adjuvant treatment significantly increased cancer cell sensitivity to cisplatin and doxorubicin. This study thus provides a potential therapeutic strategy for the treatment of endometrial cancers otherwise resistant to treatment.

## Abbreviations

ACC	acetyl-CoA carboxylase
ACLY	ATP-citrate lyase
ANOVA	analysis of variance
CHOP	C/EBP homologous protein
CI	combination index
cPARP	cleaved poly-ADP-ribose polymerase
DCFH-DA	2′,7-dichlorofluorescein diacetate
DMSO	dimethyl sulfoxide
DNL	de novo lipogenesis
ED50	median effective dose
ER	endoplasmic reticulum
FAS	fatty acid synthase
FBS	fetal bovine serum
H3P	phospho-histone H3
H3	histone H3
HO-1	heme oxygenase 1
LC3B	microtubule-associated proteins 1A/1B light chain 3B
MMP	mitochondrial membrane potential
MTT	thiazolyl blue tetrazlium bromide
Nrf2	nuclear factor-erythroid factor 2-related factor 2
PBS	phosphate-buffered saline
PI	propidium iodide
ROS	reactive oxygen species
TCA	tricarboxylic acid
